# Hepatic Amyloidosis Manifesting as Budd-Chiari Syndrome: An Unusual Presentation

**DOI:** 10.7759/cureus.76719

**Published:** 2025-01-01

**Authors:** Pritam Das, Dhruv Thakur, Gourav Jyoti Borah, Naganath K Wodeyar, Samir Mohindra

**Affiliations:** 1 Gastroenterology and Hepatology, King George's Medical University, Lucknow, IND; 2 Gastroenterology, Ganesh Shankar Vidyarthi Memorial Medical College, Kanpur, IND; 3 Gastroenterology and Hepatology, Sanjay Gandhi Postgraduate Institute of Medical Sciences, Lucknow, IND; 4 Gastroenterology, Sanjay Gandhi Postgraduate Institute of Medical Sciences, Lucknow, IND

**Keywords:** aa amyloidosis, acute budd-chiari syndrome, amyloidosis, amyloidosis with hepatic involvement, budd-chiari syndrome, gi amyloidosis, hepatic amyloidosis

## Abstract

Amyloidosis is a rare infiltrative multisystemic disorder characterized by protein misfolding, leading to progressive organ failure. It can be either acquired or hereditary. Very few case reports regarding hepatic amyloidosis with Budd-Chiari syndrome have been reported up to date. We report the case of a 45-year-old man presenting with abdominal distension, pain in the abdomen, and jaundice. Through right hepatic vein cannulation, HVPG (hepatic venous pressure gradient) was found to be 10 mmHg. The liver biopsy revealed near-total replacement of hepatic parenchyma by amorphous congophilic deposits with obliteration of sinusoids. Hepatic amyloidosis with hepatic venous occlusion is a rare entity.

## Introduction

Amyloidosis is a systemic disease that can involve the liver in primary and secondary forms (AL/AA) [1]. The extracellular amyloid deposits in hepatic blood vessels and tissues cause hepatic amyloidosis, a metabolic disease linked to systemic amyloidosis [2]. It results in an enlarged liver with a rubbery elastic consistency, known as a "lardaceous liver," disproportionate to liver enzyme abnormalities [1]. In Budd-Chiari syndrome, the hepatic vein and inferior vena cava (IVC) are blocked above the opening of the liver veins, leading to portal hypertension [3]. Hepatic amyloidosis with Budd-Chiari syndrome has been described in very few case reports, and Budd-Chiari syndrome may masquerade the diagnosis of amyloidosis. This report details a case of hepatic amyloidosis with Budd-Chiari syndrome.

## Case presentation

A 45-year-old male patient presented with complaints of abdominal distension for two weeks. The patient was initially evaluated. The ultrasound of the abdomen revealed moderate ascites and blocked hepatic veins. MR Porto venography was performed, revealing the possibility of acute Budd-Chiari syndrome. MR Porto venography reveals stenosis of the RHV (right hepatic vein), ostia of the MHV (middle hepatic vein) and LHV (left hepatic vein), intrahepatic portion of IVC, moderate hepatomegaly, and gross ascites. The patient then developed epigastric pain and yellowish discoloration of the eyes for one week. The patient also had deranged renal function and sub-nephrotic range proteinuria. The viral markers and autoimmune markers were negative.

The patient was planned for hepatic vein stenting and DIPS (direct intrahepatic portocaval shunt) if required. Under fluoroscopy and ultrasound guidance, via the right IJV (internal jugular vein) approach with 10F sheath, RHV was cannulated. The HVPG (hepatic venous pressure gradient) was measured to be 10 mmHg. The hepatic vein free pressure was 6 mmHg, and the hepatic vein wedge pressure was 16 mmHg.

**Figure 1:**
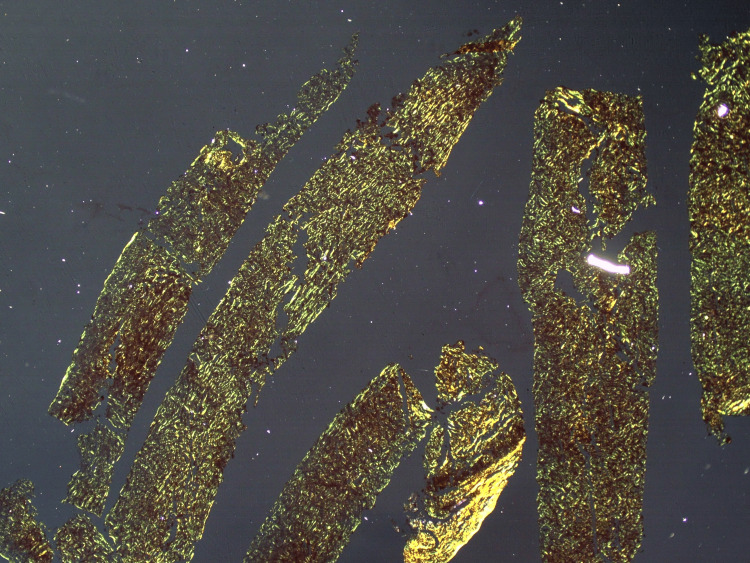
Amyloid fibrils exhibit apple-green birefringence in a liver biopsy material under polarized light microscopy.

**Figure 2:**
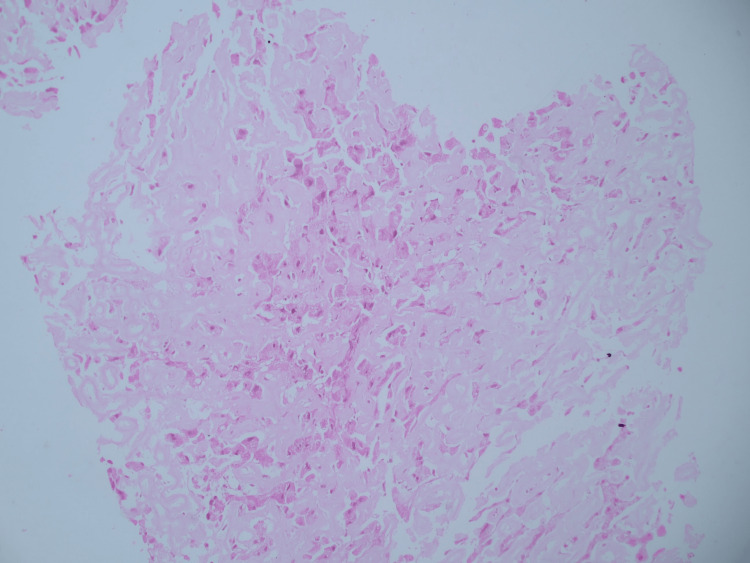
The hematoxylin and eosin stain of the liver biopsy specimen shows diffuse extracellular amyloid deposit in peri-sinusoidal spaces with compression of hepatocytes.

**Figure 3:**
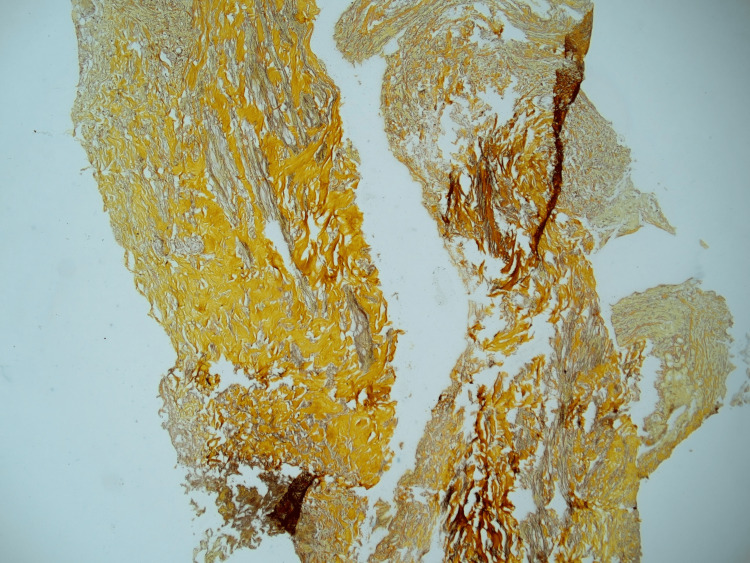
Amyloid fibrils exhibit apple-green birefringence in liver biopsy specimens under polarized light microscopy.

A liver biopsy was performed to look for a sinusoidal cause of portal hypertension. The microscopic sections revealed near-total replacement of hepatic parenchyma by amorphous congophilic deposits with obliteration of sinusoids. The congophilic deposits are also seen in the blood vessels, with few viable hepatocytes. The portal tracts show minimal portal fibrosis. The liver biopsy has been depicted in Figures 1-3.

Serum protein electrophoresis was done and revealed the absence of an M band. The prognosis regarding the outcome of the disease was explained. The patient was managed conservatively. The patient was started on diuretics and salt restriction. The ascites was mobilized. Upper GI endoscopy revealed evidence of Grade I esophageal varices. The patient was planned for a liver transplant. The initial investigations and etiological workup have been summarized in Tables 1, 2.

**Table 1:** Initial investigations at presentation S: serum; WBC: white blood cell; ALT: alanine transaminase; AST: aspartate transaminase; ALP: alkaline phosphatase

Initial Investigation	Value	Reference Range
Hemoglobin (g/dL)	13.6	13-16
WBC count (per cmm)	8400	4-10 x 10^3^
Neutrophil %	72	40-60
Lymphocytes %	24	20-40
Eosinophils %	3	1-4
Platelet count (per cmm)	3.81 L	1.5-4 L
Reticulocyte count	1.69%	-
ALT/AST (IU/mL)	24/28	5-40
ALP (IU/dL)	91	35-150
Total bilirubin (mg/dL)	0.77	1-1.3
Total protein (g/dL)	7.5	6-8
Albumin (g/dL)	4.2	3.5-5.5
Blood urea (mg/dL)	17.8	12.8-42.8
Serum creatinine (mg/dL)	0.65	0.5-1.6
S. potassium (meq/L)	4.3	3.8-5.4
S. sodium (meq/L)	130	133-146
S. calcium (mg/dL)	8.7	8.5-10.2

**Table 2:** Etiological workup IgM HAV: immunoglobulin M antibody hepatitis A virus; HEV: hepatitis A virus; HBsAg: hepatitis B surface antigen; HCV: hepatitis C virus; ASMA: anti-smooth muscle antibody; AMA: antimitochondrial antibody; LKM: liver kidney microsomal antibody; ANCA: antineutrophil cytoplasmic antibody; SPEP: serum protein electrophoresis; HVPG: hepatic venous pressure gradient; MHV: middle hepatic vein; LHV: left hepatic vein; IVC: inferior vena cava

Investigation	Value
IgM HAV	Neg
IgM HEV	Neg
HBsAg	Neg
Anti-HCV	Neg
ASMA	Neg
AMA	Neg
Anti-LKM	Neg
ANCA	Neg
ANA	Neg
SPEP	No M Band
C3	103
C4	35
Hepatic vein free pressure	6 mmHg
Hepatic Vein wedge pressure	16 mmHg
HVPG	10 mmHg
Doppler SP axis	Intrahepatic IVC stenosis with partial thrombosis MHV and LHV common orifice with focal stenosis

## Discussion

Amyloidosis is a multisystemic infiltrative disease caused by extracellular amyloid deposits in the vascular walls and tissues. The presence of light-chain immunoglobulin (AL) is a hallmark of primary amyloidosis, commonly associated with multiple myeloma, Waldenstrom macroglobulinemia, and lymphoplasmacytic lymphomas [4]. Secondary amyloidosis (AA type) is characterized by the deposition of proteins secondary to inflammation, such as rheumatoid arthritis, tuberculosis, Crohn's disease, bronchitis, and chronic osteomyelitis. Transthyretin amyloidosis (ATTR) is a hereditary or senile type of amyloid caused by mutant and wild-type transthyretins (ATTRwt) [5].

Typically, localized amyloidosis is a primary and rarely a secondary disease with amyloid material being deposited in one organ, such as the respiratory tract, skin, urinary tract, or liver. During hepatic amyloidosis, amyloid deposits are formed within the parenchyma of the liver and along the sinusoids and/or blood vessel walls, leading to hepatocyte atrophy or near disappearance [6]. There is a wide range of presentations, from hepatomegaly with borderline abnormal liver function tests to hepatic failure and portal hypertension, and rarely organ rupture [7]. The extrahepatic manifestations may include nephrotic syndrome, congestive heart failure, peripheral neuropathy, orthostatic hypotension, and hyposplenism [7].

Hepatic veno-occlusive disease is characterized by narrowing or occlusion of the hepatic lobe's central vein, causing post-hepatic portal hypertension symptoms. Swelling, degeneration, and shed endothelium were apparent in the hepatic venules, as well as proliferation and degeneration of the fibrous tissue of the vessel walls, and various degrees of stenosis were observed. There are very few sparse case reports describing Budd-Chiari syndrome in hepatic amyloidosis. The exact pathogenesis has not been elucidated. It may be attributed to a procoagulant state related to myeloproliferative disorders related to amyloidosis.

The results of liver function tests and radiological examinations are not sensitive or specific, with bilirubin and AST (aspartate transaminase) levels being normal in approximately 30% of cases. As a result of amyloid infiltration of vessel walls, areas of hypoattenuation appear in contrast-enhanced CT images, with subsequent contrast filling in delayed imaging [8]. On histological examination, amyloid appears as a homogenous extracellular material exhibiting apple-green birefringence. Upon electron microscopy, it exhibits an amorphous fibril structure, a beta-pleated sheet structure on radiographic analysis, and resistance to proteases other than pronase [9].

The treatment protocol for AL amyloidosis involves eliminating the plasma cell clone that produces the amyloidogenic light chains and achieving the lowest serum levels of the light chain involved. It will prevent further amyloid deposition and improve organ recovery [10,11]. In ATTRwt amyloidosis, the protein precursor TTR is targeted for treatment, and the patient is managed for heart failure and rhythm disorders as well. Several targeted therapies have evolved, including stabilizers and silencers [12]. AA amyloidosis should be managed by controlling the underlying inflammation that drives the amyloidogenic process. An increased mortality risk can be attributed to advanced age, elevated serum creatinine, high serum amyloid A levels, cardiac and liver involvement, and non-FMF (familial Mediterranean fever) underlying conditions [1,13].

## Conclusions

Hepatic amyloidosis manifesting as Budd-Chiari syndrome is a rare manifestation and carries a poor prognosis. The manifestation of Budd-Chiari syndrome may masquerade as amyloidosis, thereby delaying diagnosis.
